# The Challenge of AML in Older Patients

**DOI:** 10.4084/MJHID.2013.038

**Published:** 2013-06-03

**Authors:** Alan K Burnett

**Affiliations:** FMed Sci, Department of Haematology, Cardiff University School of Medicine, Heath Park, Cardiff, United Kingdom.

## Abstract

There has been a gradual improvement in the outcome for younger patients with Acute Myeloid Leukaemia over the last two decades, but unfortunately this same progress is not apparent in older patients. “Old” has come to mean rather arbitrarily, patients over 60 years. This age cut off has been perpetuated by clinical trials whose eligibility is frequently at this cut point. Age is a continuous variable right through all age groups with AML and has independent prognostic significance. Chemo-resistance of the disease itself is part of the explanation, with a high frequency of adverse biology occurring at older age. Patient characteristics which compromise the delivery of treatment of adequate intensity are the other important influence. Medical co-morbidities are more frequent, and when combined with what is sometimes referred to as limited haematopoietic reserve, undoubtedly make successful delivery of intensive therapy less likely. The outstanding problem for older patients is that remission is usually not durable, and there has been little improvement in overall survival for the last three decades, then new approaches need.

## Introduction

There has been a gradual improvement in the outcome for younger patients with Acute Myeloid Leukaemia over the last two decades, but unfortunately this same progress is not apparent in older patients. “Old” has come to mean rather arbitrarily, patients over 60 years. This age cut off has been perpetuated by clinical trials whose eligibility is frequently at this cut point. Age is a continuous variable right through all age groups with AML and has independent prognostic significance. Chemo-resistance of the disease itself is part of the explanation, with a high frequency of adverse biology occurring at older age(1,2). Patient characteristics which compromise the delivery of treatment of adequate intensity are the other important influence. Medical co-morbidities are more frequent, and when combined with what is sometimes referred to as limited haematopoietic reserve, undoubtedly make successful delivery of intensive therapy less likely. Since the median age of AML patients in western countries is around 68 years the challenge presented of treating such a sizable proportion of those with the diseases is considerable.

## Treatment Strategy

The first dilemma in older patients is whether an intensive treatment approach should be offered. A small randomised study undertaken several years ago which compared the policy of immediate introduction of standard chemotherapy with one of waiting, resulted in a similar outcome.^3^ This study was too small to come to any conclusion about which patients should be offered which approach. More recent data from the Swedish population registry indicated that patients treated in areas of the country where the approach was usually to offer intensive therapy produced a small but significant survival advantage over those regions where it was more usual to treat palliatively.^4^ In the UK AML14 trial the aim was to accrue sufficient numbers of patients to test an intensive versus non-intensive approach by randomising patients where there was uncertainty.^5^,^6^ They could be randomised to an intensive or non-intensive approach within each of which were further questions. It was hoped to have sufficient patients to enable the identification of which subgroups benefited from which approach. Over 1450 patients entered the trial but only 8 were randomised between the two treatment approaches. A multivariate analysis was carried out on the patients entering each approach. The significant factors associated with the allocation of patients were age, performance score, secondary disease, weight, and cardiac history. Interestingly when the doctor’s name was entered into the model, this turned out to be the third most important factor and also introduce both white count and marrow blast % as significant factors. Opinions vary as to whether all or any patients should be given a “3+7” approach. Others may prefer to wait for cytogenetic which will further inform the decision on the basis that patients with adverse chromosomes have little prospect of success even with standard chemotherapy. Perhaps a reasonable rule of thumb is that for patients <70 there has to be a good reason not to attempt intensive chemotherapy, and for older patients a good reason to give it. Of course in this age group the patients’ preference may make the decision. They may make an informed choice that long periods of hospitalisation which are likely to be inevitable with an intensive approach, may be too high a price to pay for a limited gain.

## An Intensive Approach

**Figure 1:**
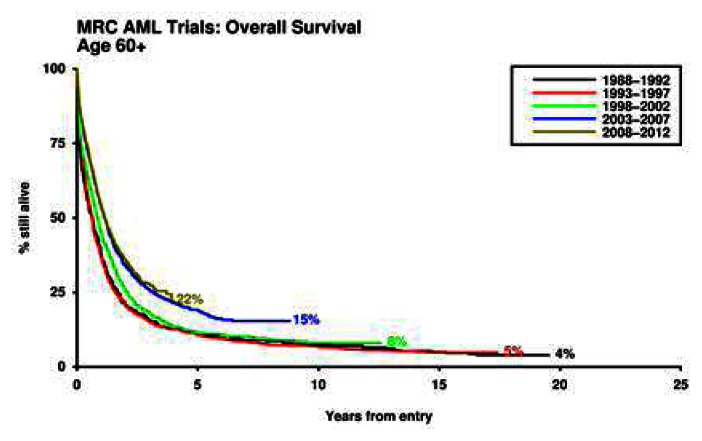
Outcome for patients over 60 years given intensive therapy

No single chemotherapy schedule has emerged as superior, in older patients, to the standard combination of daunorubicin and Ara-C. The rate of remission that it delivers varies between 40 to 65%, which is likely to be a reflection of the characteristics of the patients recruited. Exclusions because of secondary disease or inadequate renal function can make an important difference. The outstanding problem for older patients is that remission is usually not durable, and there has been little improvement in overall survival for the last three decades (Figure 1). Familiar prognostic factors (age, performance score, cytogenetic, secondary disease, presenting white count) will dictate outcome, but the stratification is less distinct than in younger patients because of the overall poorer outcome. A clear picture of the additional impact of molecular information, such as FLT3 and NPM1 mutation status is not completely clear, although similar trends to those seen in younger patients are emerging, however how much additional information they bring in the context of the other factors already mentioned is unknown. Prognostic categories as defined by the European LeukemiaNet, may be less valuable than for younger patients.^7^

**Figure 2:**
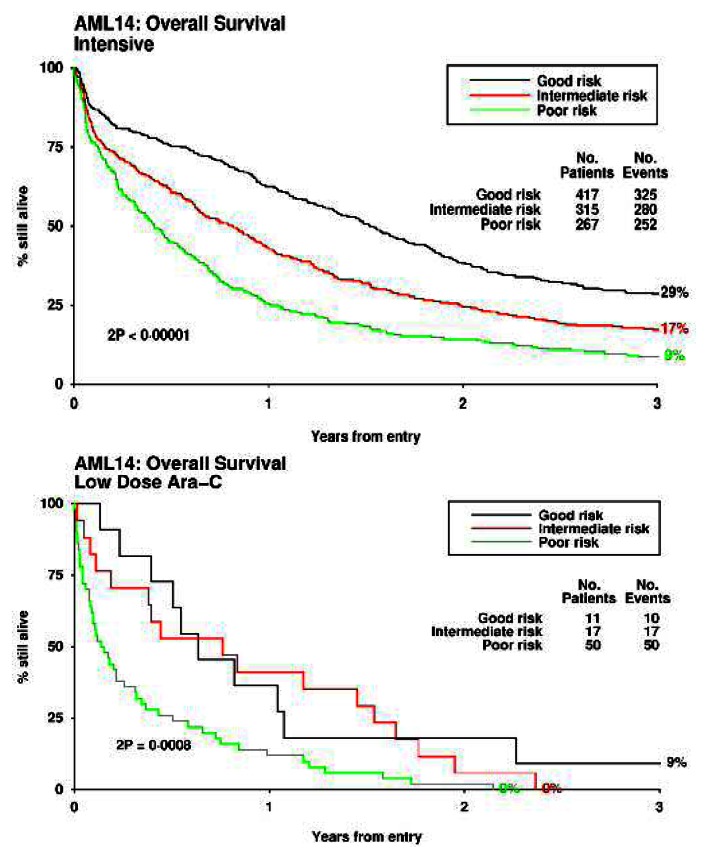
Risk score applied to patients treated intensively and non-internsively.

A number of prognostic scoring systems have been developed, some of which have been prospectively validated. Wheatley et al^8^ derived a multivariate score derived from the large MRC AML11 trial, which used a weighted score based on age, cytogenetic, secondary disease, performance score and white count, which was validated in the AML14 trial for patients treated both intensively and non-intensively. Superficial examination of the data (Figure 2) may suggest that for every category of risk, an intensive approach is always superior. However this would not be a correct interpretation because the factors beyond those included in the score may be different in patients allocated to intensive or non-intensive treatment. A similar comment could be made about what can be concluded from the Swedish Registry data.

Apart from devising a more effective induction schedule there is uncertainty about how many courses of currently available chemotherapy should be given and whether there is a role for maintenance. Maintenance using Low Dose Ara-C may have a modest benefit.^9^ Some patients may be candidates for reduced intensity allografts which appear feasible in this population although the proportion who quality is usually low partly because of donor suitability issues.^10^ As will be mentioned below, hypomethylating agents have been shown to have benefit, at least for some patients. While not achieving remission they appear to be able to keep the disease in a stable phase, which might suggest a role as maintenance.

Some of these questions are being addressed in the UK NCRI AML16 trial which completed accrual in 2012. In this trial an attempt was made to improve induction by comparing standard daunorubicin/Ara-C with daunorubicin/clofarabine. In the initial presentation of results, there was no improvement in remission rate or survival.^11^ One of the hopes from earlier unrandomised studies was that clofarabine might make a contribution in patients with adverse risk chromosomes,^12^,^13^ but this turned out not to be the case. Maintenance with the hypomethylating agent, azacitidine, for 12 months has been tested in the UK NCRI AML16 trial, but the outcome is not yet known. This is the only randomised trial of this approach, although at least one additional is planned using an oral formulation, which may achieve a more durable biological effect.

The immunoconjugate, gemtuzumab ozogamicin (GO) (Mylotarg^TM^) now only have licensed approval in Japan, but three large trials in older patients suggest that it may have a contribution to make. As part of the UK NCRI AML16 trial patients could receive daunorubicin/Ara-C or daunrubicin/clofarabine, but they were also randomised to receive GO in a modest dose (3mg/m^2^) on day 1 of induction. Although this did not make any difference to the rate of morphological remission, or minimal residual disease load in these remission marrows, it did result in a significant reduction in subsequent relapse, and since it was well tolerated, this also make a small (15% to 20%), but significant, improvement in overall survival^(14)^. A more impressive study conducted by the French ALFA Group in patients aged 50 to 70 years, similarly improved survival without altering the rate of remission.^15^ The GO was given in a similar dose (3mg/m^2^ or up to 5mg) on days 1,4 and 7 as well as in consolidation. This is likely to be more suitable for the more fit patients. The third trial adopted a different strategy, whereby patients were pre-treated with GO as single agent at a dose of 6mg/m^2^ on days 1 and 15 before proceeding to chemotherapy. This was compared with chemotherapy alone.^16^ There was no advantage in terms of remission rate or survival from this approach, indeed patients >70 years were disadvantaged.

There has been recent interest in Daunorubicin intensification, which have been supported by a number of studies.^17^–^20^ In older patients dose escalation to 90mg/m^2^ was feasible, but any survival benefit compared with a 45mg/m^2^ dose was limited to patients aged 60 to 65 years.^20^ It is not clear whether the higher dose is superior to the frequently used dose of 60mg/m^2^ or conventional doses of idarubicin. In the context of testing the P-glycoprotein modulator PSC833, the MRC AML14 trial in older patients showed no difference between a daunorubicin dose of 50mg/m^2^ and 35mg/m^2^.^5^

## A Non-Intensive Approach

There are two routes to a “non-intensive” approach. First, this may apply to patients who are considered medically fit, but who are perceived to be unlikely to benefit from an intensive approach, or where the patient has declined the intensive approach because they do not want to invest in the hospitalisation and morbidity involved for what they consider might not be a good return. Such features of the disease as age, adverse cytogenetics, secondary disease or some validated risk score can illuminate this choice. This immediately raises controversy, until it has been proven that there are alternative treatments in the non-intensive options which are superior. The second route is where there is genuine concern that intensive therapy may prove too toxic and threaten to shorten life. While most haemato-oncologists recognise this category of patients by the “end of the bed” test, and this choice is less controversial, such patients cannot be 100% reliably objectively identified. Age, patient co-morbidy and performance assessment scoring can be used. In the context of comparative clinical trials it is useful in such patients to document why a patient was not treated intensively. This may have a component of doctor bias as discussed above.

## The Options

In the past non-intensive management meant supportive care with transfusion and antibiotics as required, with a minimal amount of hospitalisation combined with intermitted chemotherapy agents such as hydroxyurea to control the white count. Although there have always been anecdotal experiences of such a strategy working in individuals for months, in general this results in a survival of 2–3 months. The UK AML14 trial compared this approach to regular courses of Low Dose Ara-C (LDAC) given a 20mg subcutaneously twice a day for 10 days administered at 4–6 week intervals.^6^ The trial randomised 201 patients with a median age of 76, but was prematurely stopped by the Data Monitoring Committee because of a superior survival in the LDAC arm. Since this was the first trial attempting to develop new approaches for this patient group it could be regarded as establishing a new “standard of care”. However while it may be a standard of care, it is far from satisfactory. The survival benefit was confined to the 18% of patients who entered complete remission who enjoyed a median duration of CR of 15 months. No patient in this study with adverse cytogenetics entered remission and so the preliminary observation was that this approach was not beneficial for this important subgroup. In subsequent experience however there has been a remission rate of about 11%, so it is incorrect to conclude that there is absolutely no benefit in this subgroup.^21^,^22^ One reservation about the LDAC approach was that it would simply add toxicity without benefit for more than a few patients, but the AML14 trial showed no difference between the arms in this respect. It is therefore reasonable to conclude that “best supportive care” should no longer be considered an option for these patients.

## Alternatives to LDAC

While LDAC should displace BSC, it remains a far from satisfactory treatment, so improvement is urgently required. Randomised trials to achieve this are few. Demethylation agents are considered by many as a useful approach for this patient group. However the randomised evidence bears closer examination. Azacytidine is approved for high risk myelodysplastic syndrome where it delays the transformation to AML provided a sufficient number of courses are given. Included in the pivotal trial (ADZ001) were a population of 110 patients with marrow blasts between 20 and 30% which at the time were not yet classified in the WHO definition as AML.^23^ However in the current definition these patients are classified as AML. While this move could be debated, a retrospective examination of the UK database showed no survival difference in older AML patients with marrow blast greater or less than 30%. However in this subset within the ADZ001 trial, azacytidine resulted in a significant improvement on overall survival, without improving the remission rate, compared with the control patients who were treated by “doctor’s choice”.^23^ This lead to the regulatory approval of azacytidine for this patient subset. Commendably, however, the trial required participating physicians to pre-specify before randomisation what treatment each individual patient would receive as BSC comparison. While there was a trend for benefit when compared with the LDAC group, the difference did not reach significance. A further important reservation about the evidence from this trial is the observation that the recipients of BSC had a median survival of approximately 15 months. This is not typical of AML patients (even in the 20–30% subset) treated with BSC suggesting that the patients recruited to this MDS trial were not true AMLs. The LDAC versus azacytidine competition will hopefully resolved by the on-going AZA-AML-001 trial in patients with >30% blasts which is now fully recruited, although the comparator arm was again “doctor’s choice”.

A second demethylator, decitibine, is frequently chosen for these patients. The randomised evidence was obtained from the CCO 2009 trial^24^ with compared decitabine with LDAC with overall survival being the primary endpoint. The regulatory submission intended the primary analysis to be carried out after a certain number of events, and although there was an approximate 2 month median difference in favour of decitabine, this did not reach significance. However in a follow up analysis this difference became significance. Another observation was that the LDAC given in the control arm was only given once a day rather than the twice a day standard. This resulted in a CR rate of only ~8%, this may or may not be important because the median survival of the LDAC arm at 5 months was as expected. Recruits to this trial were required to have acceptable renal function and since renal function can have a negative impact in this patient group, this may represent an unintentional selection.

While there are some patients, perhaps with less proliferative disease, for whom demethylation therapy is advantageous, the challenge is how to identify them at diagnosis. This experience however also challenges the dogma that the only route to improving survival is to improve the remission rate, which raises the question of whether demethyaltion could be a useful strategy in other circumstances such as maintaining remission.

## Novel Approaches

Many drugs have failed to gain approval in AML not least because the target has been advanced disease where there may be no consensus on the standard of care and the disease is more refractory. The setting of the unmet need in the older “unfit” population has become an attractive setting. However apart from the demethyation agents discussed above nothing has yet achieved regulatory approval. Given the inadequacy of current therapy and the likely expense of new agents it is reasonable to review strategy with respect to trial design. The objective is to find treatments that will make a *clinical* difference i.e. not a few percentage points in survival, thus allowing trials to require fewer patients. This, for example, may require that a new drug is expected to double the response rate and 12 month survival. This approach might miss an agent which has a modest benefit, but will be more efficient in eliminating treatments which are unlikely to be useful. Another difficulty that has to be taken into account is the administrative delay in setting up a new trial. In the UK (and collaborating countries) such an approach is being adopted in a “Pick a Winner” trial design whereby novel treatments are tested against “standard of care” (LDAC).^25^ This design requires contemporaneous randomisation and uses an early endpoint (achievement of remission) as a surrogate for benefit. If this is not likely to be doubled the treatment is abandoned. For certain drugs such as demethylation agents this may not be suitable, in which case the judgement can be made on the outcome at 12 months. The details of this approach has been set out elsewhere.

### Clofarabine

This novel nucleoside showed interesting efficacy in relapsed disease, but ultimately failed in a randomised trial in combination with Ara-C, with overall survival as the endpoint. At lower doses it turned out to be well tolerated in older patients in two trials with encouraging efficacy in all groups, but these studies were not randomised. There was remarkable consistency of effect such as delivering remissions in >40% of patients irrespective of age or cytogenetic group. In the Pick a Winner Programme it passed initial assessment by doubling the remission rate and so was expanded to randomise >400 patients versus LDAC. Disappointingly, survival was not improved overall or in any subgroup.^21^

### The Addition of Arsenic Trioxide

An unrandomised phase 2 study conducted by Roboz^26^ and colleagues explored the addition of arsenic trioxide to LDAC therapy. Encouragingly this achieved a remission rate of nearly 40%, thus becoming a combination worth further study. However, when tested in a randomised setting versus LDAC it failed to fulfil the initial criteria for continuation in the Pick a Winner Programme, and therefore did not continue.^27^

### Tipifarnib

Mechanistically a case can be made for farnesyl inhibition in AML. This was tested in AML in the treatment of relapsed disease where the second remission rate was disappointing at 6%.^28^ More encouraging was the experience as monotherapy in the older unfit untreated population where a remission rate similar to LDAC was seen.^29^ This unrandomised experience was insufficient to achieve regulatory success, but triggered interest in a combined approach with LDAC. Again when subjected to randomised comparison versus LDAC alone no benefit was seen.^22^ This drug remains of interest in combination with chemotherapy or as maintenance.

### Gemtuzumab Ozogamicin

This immunoconjugate intended to deliver chemotherapy by targeting the CD33 epitope has had a turbulent history. This included the possibility of being effective as monotherapy in the untreated older patient with modest success. It significantly improved the CR in combination with LDAC versus LDAC alone in the Pick a Winner Programme, but like the experience with clofarabine failed to improve the survival in the expanded study.^30^

### Polo like Kinase Inhibitor (Volasertib)

Very recently presented data suggested that this agent in combination with LDAC may be superior that LDAC alone. In a preliminary randomised trial the marrow remission rate was better with the combination (31% vs 13%), and there was a significantly superior disease free survival, but not yet overall survival.^31^ This has paved the way for an ambitious large randomised trial which is due to commence in 2013.

### Other Agents

several new agents are at an early stage of development in AML which could be candidates for evaluation in this older patient population. The risk is that these treatments might in some cases be almost as intensive as standard chemotherapy. Another novel nucleoside, sapacitibine, has shown efficacy in older patients with relapsed disease^32^ and is being directly compared with LDAC in the Pick a Winner Programme. Vosaroxin, a novel topo II anthracycline-like drug^33^ with is not susceptible to P-glycoprotein or p53 mediated resistance, clearly has efficacy as monotherapy in relapsed or refractory disease, but based on preclinical evidence of synergy, there also a rationale of assessing it in combination with LDAC. This may be an example of a drug which results in a similar level of myelosuppression as conventional 3+7 chemotherapy.
